# The Influence of Insertion Torque on Stress Distribution in Peri-Implant Bones Around Ultra-Short Implants: An FEA Study

**DOI:** 10.3390/jfb16070260

**Published:** 2025-07-14

**Authors:** Mario Ceddia, Lorenzo Montesani, Luca Comuzzi, Alessandro Cipollina, Douglas A. Deporter, Natalia Di Pietro, Bartolomeo Trentadue

**Affiliations:** 1 Department of Mechanics, Mathematics and Management, Polytechnic University of Bari, 70125 Bari, Italy; bartolomeo.trentadue@poliba.it; 2 Independent Researcher, 00131 Rome, Italy; lomonte@bu.edu; 3 Independent Researcher, 31020 San Vendemiano, Italy; luca.comuzzi@gmail.com; 4 Independent Researcher, 92019 Sciacca, Italy; alexandros1960@libero.it; 5 Faculty of Dentistry, University of Toronto, Toronto, ON M5G 1G6, Canada; douglas.deporter@dentistry.utoronto.ca; 6 Department of Medical, Oral and Biotechnological Sciences, University of Chieti-Pescara, 66100 Chieti, Italy; natalia.dipietro@unich.it

**Keywords:** insertion torque, ultra-short dental implants, finite element analysis, atrophic jaws

## Abstract

Using ultra-short dental implants is a promising alternative to extensive bone grafting procedures for patients with atrophic posterior mandibles and vertical bone loss. However, the amount of insertion torque (IT) applied during implant placement significantly influences stress distribution in the peri-implant bone, which affects implant stability and long-term success. Materials and Methods: This study used finite element analysis (FEA) to examine how different insertion torques (35 N·cm and 75 N·cm) affect stress distribution in cortical and trabecular bone types D2 and D4 surrounding ultra-short implants. Von Mises equivalent stress values were compared with ultimate bone strength thresholds to evaluate the potential for microdamage during insertion. Results: The findings demonstrate that increasing IT from 35 N·cm to 75 N·cm led to a significant increase in peri-implant bone stress. Specifically, cortical bone stress in D4 bone increased from approximately 79 MPa to 142 MPa with higher IT, exceeding physiological limits and elevating the risk of microfractures and bone necrosis. In contrast, lower IT values kept stress within safe limits, ensuring optimal primary stability without damaging the bone. These results underscore the need to strike a balance between achieving sufficient implant stability and avoiding mechanical trauma to the surrounding bone. Conclusions: Accurate control of insertion torque during the placement of ultra-short dental implants is crucial to minimize bone damage and promote optimal osseointegration. Excessive torque, especially in low-density bone, can compromise implant success by inducing excessive stress, thereby increasing the risk of early failure.

## 1. Introduction

Significant vertical bone loss frequently occurs in the posterior regions of the mandible, particularly in patients who have not undergone prolonged rehabilitation or who have used removable prostheses for extended periods [1,2,3]. According to the literature, an average reduction of 1–3 mm can occur within the first six months following tooth extraction, with resorption potentially continuing at a gradual rate of several millimeters per year over time [4,5,6,7]. The stable placement of conventional dental implants presents significant challenges in cases of a markedly reduced bone volume.

Bone grafting is one of the most common strategies used to address reduced bone availability during implant rehabilitation. However, numerous studies and clinical observations have highlighted several critical limitations and potential adverse effects of this procedure that must be carefully considered during treatment planning [8,9,10]. First, bone grafting interventions are more surgically invasive than implant placement alone [11,12,13,14,15]. This exposes patients to an increased risk of intraoperative and postoperative complications, such as infection, graft rejection, and delayed soft tissue healing [16]. Furthermore, bone derived from grafting procedures may have lower density and poorer biomechanical properties than native bone. This can result in an increased susceptibility to marginal bone resorption, as reported by Esposito et al. [17]. This condition is particularly critical under high masticatory loads since lower bone quality impairs the uniform distribution of mechanical forces. This can lead to overload and subsequent biological and prosthetic complications [18,19].

In recent years, short and ultrashort implants have gained attention due to their improved design and efficacy [16,17,18,19,20]. These implants provide a viable solution for managing atrophic alveolar ridges and minimize the need for invasive surgical procedures and their associated risks. Furthermore, clinical studies have demonstrated that their long-term success rates are comparable to those of conventional implants [20,21,22,23]. There is no definitive consensus on the thresholds that define short or ultrashort dental implants; however, this study adopted the classification proposed by Al-Johany et al. [24], which defines ultrashort implants as those measuring 6 mm or less in length. More recently, Lombardo et al. [25] defined “ultrashort” dental implants as those measuring 5 mm or less in length. These researchers reported a 96.6% survival rate for single crowns on short and ultrashort implants after three years.

Pistilli et al. [26] documented an absence of implant or prosthetic failure in a clinical case involving 4-mm implants over a seven-year follow-up period. Felice et al. [23] found that, five years after loading, short implants exhibited significantly lower marginal bone loss compared to standard-length implants. Implant success depends on multiple factors, including implant geometry, loading conditions, and bone quality. Additionally, how forces are transferred to the surrounding bone is a key element that significantly impacts implant success [27,28,29,30,31,32,33]. Excessive forces and stress can lead to crestal bone loss through osteocyte apoptosis, which triggers signaling pathways that recruit osteoclasts responsible for bone resorption [34].

The insertion torque applied during implant placement is critical to achieving biological acceptance by the surrounding bone. Clinical observations have demonstrated a direct relationship between insertion torque, speed, and the success rate of implants [35]. In a randomized controlled clinical trial, Amari et al. [36] compared implants placed with extremely low insertion torque (less than 10 N·cm) with those placed with standard torque (around 30 N·cm). The study reported that there was more newly formed bone in contact with the implant surface at sites with higher torque and a higher implant failure rate at sites with lower torque. However, there is no universally recommended insertion torque value that can ensure implant success. For example, Trisi et al. [37] demonstrated that high insertion torque (mean 110 N·cm) did not induce bone necrosis but enhanced primary implant stability.

In contrast, a randomized clinical trial by Alfonsi et al. [38] found that high tightening torques (50–100 N·cm) in healed ridges could lead to increased peri-implant bone remodeling and soft tissue recession after two years compared to implants placed with lower insertion torques (less than 50 N·cm). Yet, Ruppin et al. [39] reported no statistically significant differences in marginal bone loss during the first three months of healing between implants inserted with high torque (80 N·cm) and those inserted with regular torque (50 N·cm).

A key factor in optimizing insertion torque is bone density because adequate bone density enables sufficient torque to ensure primary stability, which is essential for osseointegration and long-term implant success [40]. Excessively high torque in dense bone can cause damage, such as necrosis or marginal resorption, while insufficient torque in poor-quality bone may fail to provide adequate stability, increasing the risk of implant failure [41]. In summary, the ideal insertion torque should be tailored to bone quality and density. This balances the need for adequate primary stability with the risk of bone trauma. Thus, it creates optimal conditions for stable and durable osseointegration.

Finite element analysis (FEA) has recently enabled the evaluation of stresses occurring in dental implants and the surrounding bone [42,43,44,45]. A significant advantage of FEA is its ability to analyze the numerous factors that influence the success of implant treatments in a non-invasive manner. However, few studies have investigated stress distribution in the crestal and trabecular regions during short implant insertion across different bone densities [45,46,47,48,49,50]. It is crucial to achieve an optimal stress profile during this process to protect the surrounding bone. Excessive stress can cause irreversible bone damage and lead to implant failure, while insufficient stress may not adequately stimulate healing and osseointegration. Therefore, the aim of this study was to analyze stress in the cortical and trabecular bone regions during short implant insertion. This study evaluated the effects of two insertion torque levels (35 N·cm and 75 N·cm) on two bone quality classes (D2 and D4). This analysis will provide insight into the optimal insertion torque for placing short implants in different bone densities.

## 2. Materials and Methods

Three-dimensional (3D) models were created using Auto Desk Inventor 2024 to analyze the distribution of stress during implant placement. Finite element simulations were conducted in ANSYS Workbench R 2023. A 3D representation of a mandibular bone segment was generated from computed tomography (CT) scans of a healthy, posterior, edentulous mandible. The geometry was reconstructed in ANSYS Space Claim based on CT-derived contours [51,52,53].

**Figure 1:**
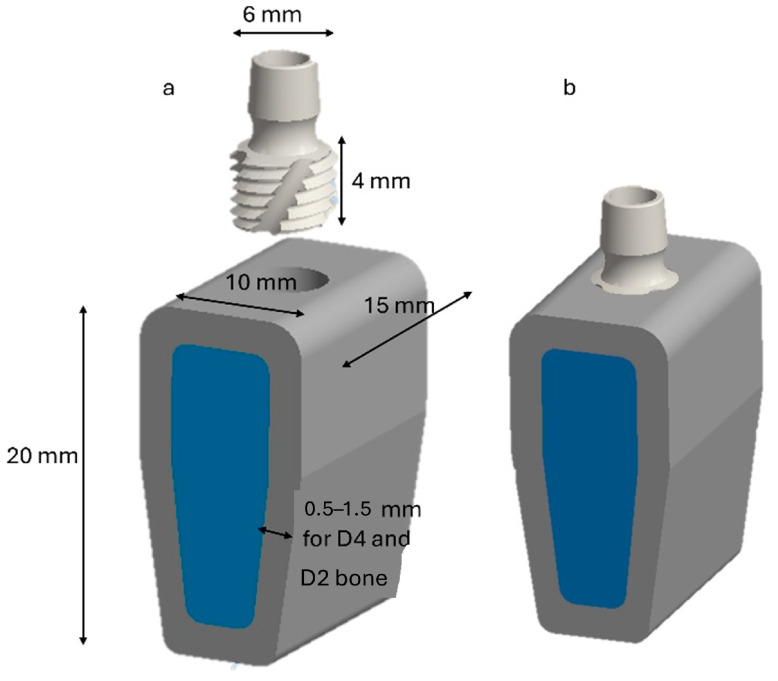
Three-dimensional (3D) modeling. (**a**) Details of the bone model and implant; (**b**) model with the implant inserted in the crestal position.

The modeled segment was prismatic and block-like, adapted to follow the natural curvature of the mandibular ridge. Its approximate dimensions were 20 mm in vertical height (from the mandibular base to the alveolar crest), 15 mm in mesiodistal width (along the dental arch), and 10 mm in buccolingual width at the crest’s apex. Modeling of the cortical layer was performed by creating a uniform shell with thicknesses of 1.5 and 0.5 mm on the superior surface and lateral aspects (buccal and lingual), respectively, in accordance with established anatomical data. The structure consisted of trabecular bone with a gradual decrease in density toward the basal region, reflecting the transition from D2 to D4 quality [54]. Figure 1 shows a graphical representation of the modeled geometry.

An ultra-short implant measuring 6 mm in diameter and 4 mm in length (AoN Implants Srl, Grisignano di Zocco, Italy) was used and inserted at the crest of the bone model (see Figure 1b). The bone hole was created by subtracting the size of the implant from the bone model. This represents an ideal situation of perfect adhesion between the bone and the implant but does not occur in clinical reality. A classification system was employed in this study for bone structures and mechanical properties, as proposed by Misch et al. [55]. This classification comprises four classes: D1 to D4. Class D1 is the densest class, consisting almost entirely of cortical bone tissue. It is mainly found in the anterior part of the mandible. In contrast, class D4 consists of low-density trabecular tissue and is found in the posterior part of the mandible.

According to Pammer et al. [56], the mechanical properties of the D2 and D4 bones were based on data from the literature used to model different bone classes in a finite element analysis. However, bone tissue behaves differently in different directions due to its anisotropic nature, which is determined by its structure. The anisotropic behavior of cortical bone stems from the orientation of collagen fibers and the arrangement of bone lamellae. Bone lamella strengthens the bone along the direction of the fibers themselves [57].

In trabecular bone, anisotropy stems from the orientation of the trabeculae along lines of force. This directionality enables the bone to withstand stresses better in the preferred directions while being less resistant in other directions. Variations in trabecular orientation and density greatly affect the mechanical response of bones under different loading conditions and during processes such as implant insertion [58].

A study by Taheri et al. [59] showed that isotropic models underestimate stress values in bone tissue. Furthermore, Gasik et al. [60] developed numerical models that incorporate heterogeneous bone density obtained from CT imaging. These models demonstrate that anisotropic models dependent on bone density produce higher and more realistic stress distributions than homogeneous models. Therefore, modeling a bone as an anisotropic material provides a more accurate representation of its actual mechanical response under load. Since it is difficult to obtain mechanical properties in different directions, several researchers have correlated bone sample density with mechanical properties, such as stiffness, which is described by Young’s modulus (E) and Poisson’s ratio (ν) [56,61,62,63,64,65]. These researchers obtained relationships such as those reported in Equations (1) and (2).

(1) $$E_{cancellous}=1.904\times \rho^{1.64}$$

(2) $$E_{cortical}=2.065\times \rho^{3.09}$$

At this point, Equations (1) and (2) allow us to derive the corresponding mechanical properties of a bone, assumed here to be isotropic, as shown in Table 1 [56].

Density, ρ Poisson’s ratio, vThe implant was made of Ti-6Al-4V titanium alloy, and its mechanical properties are shown in Table 2 [56,57,58,59,60,61].

### 2.1. Boundary and Load Conditions

**Figure 2:**
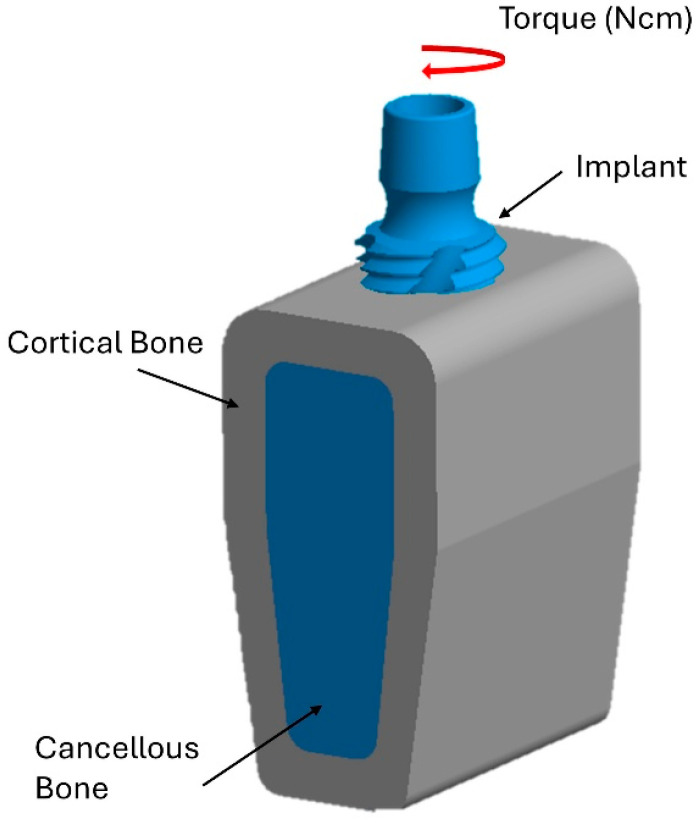
Application of insertion torque on the upper surface of the implant.

The bottom surface of the bone block was fixed in all directions. Additionally, the contact interaction between the bone and the implant was defined using the friction contact function available in Ansys, with a friction coefficient set at 0.35 [66]. The implant was inserted into the model with a rotational movement at a speed of 20 rpm applied to the upper part of the implant, simulating the screwing procedure during actual implant insertion. This rotation creates dynamic stress on the surrounding bone. In accordance with the clinical protocol of the implant manufacturer, the insertion torques considered were 35 N·cm and 75 N·cm (see Figure 2).

### 2.2. Simulation of the Insertion Technique

**Figure 3:**
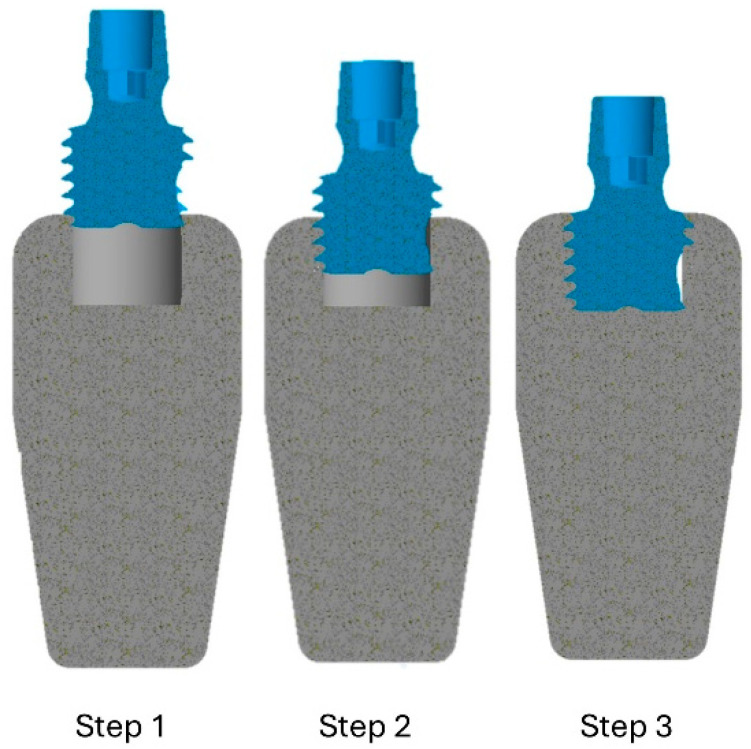
Details of the three analyzed insertion steps in this study for D2 and D4 bone types with two insertion torque levels of 35 N·cm and 75 N·cm.

This study, conducted in collaboration with dental and engineering experts, aimed to dynamically simulate the process of inserting dental implants. Due to the complexity of modeling continuous insertion, a stepwise approach was adopted to simplify the process. Three finite element models were created, each representing an implant inserted into D2 and D4 bone blocks. These models progressively covered the entire 4 mm length of the implant. Initially, the implant was inserted 0.2 mm into the cortical bone. As the insertion depth increased, the bone–implant contact area significantly expanded, altering the stress distribution pattern within the mandible (Figure 3). This simulation approach enabled a detailed analysis of stress variations and biomechanical interactions during the various stages of implant insertion.

### 2.3. FEA Modeling

**Figure 4:**
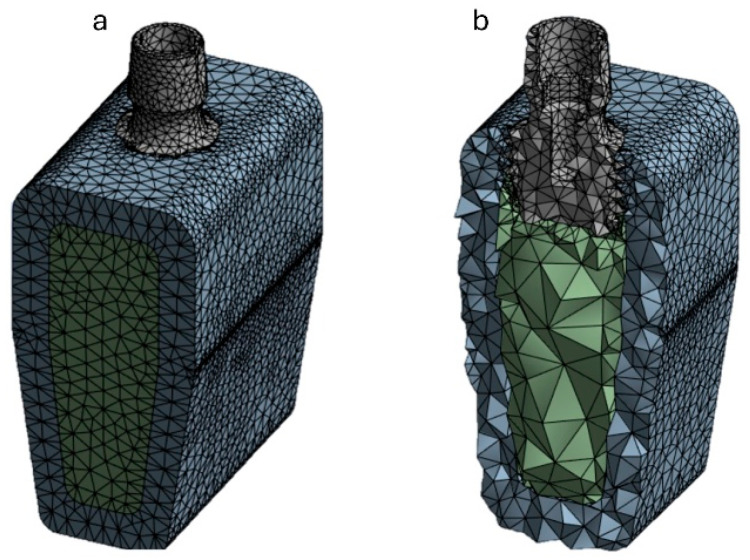
Discretized model of the bone block and implant. (**a**) Complete model; (**b**) sectional view showing the tetrahedral elements.

The finite element modeling (FEM) of the implant and mandible was performed using Ansys Workbench 2023 R1. The 3D geometry was discretized with 10-node quadratic tetrahedral elements (SOLID 187), which are suitable for handling complex contours and provide accuracy in calculating stress gradients. Using a 0.5 mm mesh size, the bone model was discretized into approximately 85,000 solid elements, while the implant model used about 31,000 elements of the same type (see Figure 4). A mesh convergence test was conducted to balance the accuracy of the results and computation time to obtain a more accurate estimate of the local damage caused by implant insertion. Local mesh refinement was applied in the contact area between the bone and the implant. Specifically, a 0.2 mm mesh was used at the implant threads. The variations in the results were less than 5%, indicating that the chosen mesh size represents an optimal compromise for this study.

## 3. Results

During the insertion of a dental implant, the bone material is subjected to stresses that may exceed its yield strength. This can result in localized plastic deformation and potential microfractures. Therefore, stress analysis focuses on von Mises equivalent stress, a parameter commonly used to predict the elastoplastic behavior of isotropic, homogeneous materials. In the present study, von Mises equivalent stress values obtained through finite element analysis were compared with the respective ultimate strength values of cortical bone (90 MPa) and cancellous bone (10 MPa) [67,68]. This comparison allows us to evaluate the risk of bone damage during implant insertion and optimize the procedure to ensure stress remains within physiological limits conducive to successful osseointegration.

### 3.1. Analysis of Stress in Bone D2

**Figure 5:**
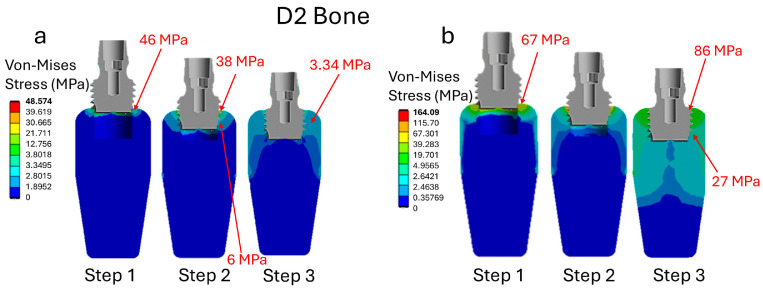
Stress analysis during implant insertion for a D2 bone type. (**a**) Insertion torque 35 N·cm; (**b**) insertion torque 75 N·cm.

The analysis of the D2-type bone reveals a maximum stress value of 46 MPa associated with an insertion torque of 35 N·cm, as shown in Figure 5a. This maximum stress is concentrated primarily on the cortical bone during the initial phase of implant insertion (Step 1). As the implant advances within the bone, cortical bone stress decreases, reaching approximately 38 MPa. Meanwhile, stress on the trabecular bone increases, peaking at 6 MPa. Once the implant is fully inserted (step 3), stress tends to equalize between the two bone types, reaching similar values of around 3.34 MPa in both the cortical and trabecular bones. With a higher insertion torque of 75 N·cm (Figure 5b), a significant increase in stress is recorded, peaking at 67 MPa on the cortical bone by step 1. Additionally, during the initial insertion phase, stress is transmitted to the trabecular bone. Throughout the entire procedure, stress values remain higher than in the lower torque case. At the end of insertion (step 3), the stress on the cortical bone is still high, reaching 86 MPa, while the stress on the trabecular bone is 27 MPa.

These data suggest that a significant increase in insertion torque results in a substantial rise in stress on cortical and trabecular bone. Elevated stress levels, particularly in the cortical bone, can exceed the bone’s capacity. This can potentially cause microdamage, increased bone resorption, and impaired healing. These adverse effects compromise bone integrity and may negatively impact the long-term success and stability of the dental implant. Therefore, controlling insertion torque is critical to minimizing bone damage and promoting optimal osseointegration to enhance implant longevity.

### 3.2. Analysis of Stress in Bone D4

**Figure 6:**
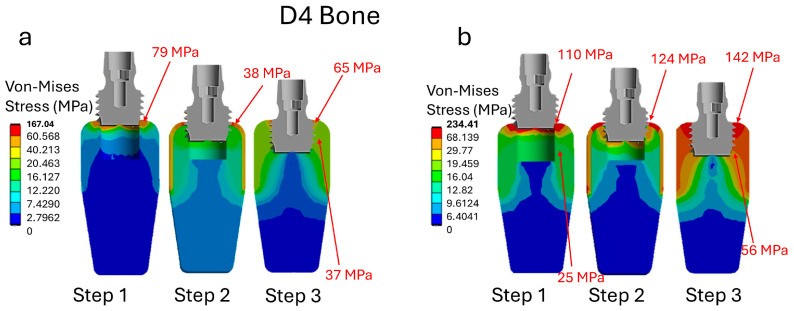
Stress analysis during implant insertion for a D4 bone type. (**a**) Insertion torque 35 N·cm; (**b**) insertion torque 75 N·cm.

Figure 6a shows that with an insertion torque of 35 N·cm during Step 1 (initial insertion), the maximum stress in the cortical bone is approximately 79 MPa. As the implant progresses to Step 3 (fully inserted), the maximum stress in the cortical bone decreases to approximately 65 MPa, while the maximum stress in the trabecular bone is 37 MPa. The reduction in cortical bone stress during insertion is due to the fine architecture of the trabecular bone, which absorbs a large portion of the mechanical load. This reduces the stress transmitted to the less resistant cortical bone in the D4 bone quality. When the insertion torque increases to 75 N·cm in the D4 bone (Figure 6b), significant stress increases are observed from the initial stages of implant insertion. Specifically, cortical bone stress begins at approximately 110 MPa in Step 1, rises to 124 MPa in Step 2 (the intermediate phase), and reaches 142 MPa in Step 3. Concurrently, the stress in the trabecular bone rises from 25 MPa to 56 MPa over the same period. These elevated stress levels indicate a substantially higher mechanical load on the bone, which may increase the risk of bone damage and negatively affect the implant’s long-term success. These values are significant because they approach the ultimate strength limits of cortical and trabecular bone tissue.

According to the literature, the ultimate strength is about 90 megapascals (MPa) for cortical bone and 10 MPa for trabecular bone [69,70,71]. Exceeding these critical stress thresholds indicates an elevated risk of mechanical damage, such as microfractures or bone necrosis, which can compromise primary stability and osseointegration. Specifically, the D4 bone characteristics are defined by a fine trabecular structure and lower bone density. This results in a markedly limited load-bearing capacity, making the tissue more vulnerable to excessive stress. Therefore, a high insertion torque of 75 N·cm in the D4 bone results in stresses that surpass those at a lower torque of 35 N·cm and exceed the bone’s strength limits. This increases the risk of long-term complications, including marginal bone loss and implant instability. These findings highlight the importance of carefully regulating insertion torque in low-quality bone. They also suggest the potential benefits of using surgical techniques or instruments, such as cortical drills or bone densification methods, to reduce the risk of tissue damage and improve implant success [72,73,74,75,76].

## 4. Discussion

Of late, short and ultrashort implants have gained attention as less invasive alternatives to complex bone augmentation procedures [77,78,79,80].

These implants offer several advantages, including reduced invasive surgery, shorter healing periods, lower cost, and improved patient comfort. According to the literature, 4 mm implants have comparable survival rates to longer implants and result in reduced marginal bone loss and fewer biological and prosthetic complications [81].

Current systematic reviews and meta-analyses confirm that short implants are a reliable option for treating posterior maxillary atrophy without resorting to invasive augmentation procedures.

In recent years, there has been a growing trend of reducing implant length further, and ultra-short and extra-short implants of 5–6 mm or less are now commercially available. These implants have been shown to achieve osseointegration and support functional loading [82,83,84]. For instance, an in vitro study by Moreno et al. demonstrated that short implants yield insertion torque (IT) and primary stability values like those of conventional implants. Similarly, Comuzzi et al. [85] reported that short and ultra-short implants provide adequate primary stability. Short implants generally perform better in resonance frequency analysis (RFA), while ultra-short implants demonstrate promising insertion torque (IT) values, particularly in denser bone.

Clinically, increasing insertion torque is often used to improve primary stability. However, this can result in irreversible damage to the cortical bone, which can lead to bone loss and early implant failure. Additionally, the stress generated around the implant is difficult to measure in clinical settings.

Finite element analysis (FEA) is a widely used method in science and industry for evaluating stress and strain in complex systems, such as the bone–implant interface [86,87]. FEA simulates complex physical systems and offers approximate numerical solutions describing their response to applied loads. FEA has become a powerful tool for predicting the biomechanical performance of various implant designs and evaluating how clinical factors influence implant success, as direct measurement of stress in vivo is difficult. Numerous studies have examined the geometry of implants (e.g., length and diameter) and the biomechanical interactions between implants and their components. These studies have primarily focused on stress distribution in bone and materials after placement, mostly using static FEA models [82,83,84,85,86,87,88]. Understanding stress transmission during implant insertion is essential because bone stress directly impacts long-term success.

Excessive stress, particularly on the marginal cortical bone, can cause mechanical damage, such as microfractures and thermally induced necrosis. This can ultimately lead to bone resorption and early implant failure [89,90,91]. Conversely, insufficient stress may fail to stimulate adequate bone remodeling, which can compromise healing, repair, and osseointegration. Thus, accurately managing and understanding the stress profile during implant insertion is critical to preventing damage, promoting primary stability, and ensuring proper healing and long-term integration.

In a dynamic finite element analysis (FEA) study by Demirbas et al. [92], the application of high insertion torques, specifically 75 N·cm in the D4 bone, resulted in stress values that exceeded the reported resistance thresholds in both cortical and trabecular tissues. This increase in mechanical load was associated with a higher risk of damage, including microfractures and bone necrosis. The study also demonstrated that using a cortical drill significantly reduced stress and potential damage in the crestal region without compromising primary stability.

Similarly, Van Staden et al. [93] used finite element analysis (FEA) to evaluate stress profiles within the mandibular bone during implant insertion. Their research revealed that stress levels in cortical and trabecular bone fluctuate based on insertion depth and applied torque. Peak stress values were observed near the implant neck during the final stages of insertion. These results support the established principle that insertion torque is directly proportional to the stress transmitted to the peri-implant bone. Furthermore, Joshi et al. [94] conducted a review that highlighted bone density as a critical factor in determining the appropriate insertion torque during dental implant placement. As numerous studies have reported, the D1 bone, which is characterized by high density and compactness, allows for higher insertion torque values. These higher values enhance primary stability and lead to improved implant success rates. In contrast, lower-quality bone types, such as D3 and D4, which are commonly found in the posterior maxilla, are associated with less predictable torque responses and a higher incidence of failures or complications. To mitigate the risk of implant or bone damage, many studies have recommended a maximum insertion torque limit of 80 N·cm across all procedures.

However, the maximum and minimum thresholds for insertion torque depend on several factors, including bone density and implant geometry. For example, implants with larger diameters or greater surface contact areas (e.g., longer implants) typically require higher insertion torque values because they provide greater mechanical resistance during placement. Based on this observation, several studies have used finite element analysis (FEA) to evaluate the impact of insertion torque on bone stress when placing standard-length implants [95,96,97]. Conversely, few studies have focused on short implants specifically and their mechanical performance in relation to different bone quality classes.

This study analyzed the influence of insertion torque on the distribution of peri-implant stress around ultra-short dental implants using an FEA model. The results indicate that increasing insertion torque from 35 N·cm to 75 N·cm significantly increases stress levels in both cortical and trabecular bone. This increase has potential negative implications for bone integrity and the long-term success of the implant, particularly in D4-type bone. These findings are consistent with previous studies reporting that excessive insertion loads may lead to mechanical damage to the cortical marginal bone, such as microfractures and thermally induced necrosis due to mechanical trauma and elevated temperatures [97,98,99,100,101]. These phenomena are commonly associated with marginal bone loss and early implant failure. Conversely, insufficient insertion torque may not provide adequate primary stability, thereby compromising healing and optimal osseointegration.

It is important to note that an increase in insertion torque does not necessarily result in bone damage or resorption. If the stress induced by the torque remains below the tissue’s mechanical strength, the bone may exhibit a form of biological self-protection by dynamically adapting to mechanical stimuli. This adaptive response involves remodeling processes that compensate for the effects of high loads, preserving the tissue’s structural integrity. This mechanism may explain why a bone does not undergo atrophy in many clinical situations despite theoretically elevated stress levels, and why implants continue to function successfully. A key strategy to mitigate these risks is to use more conservative and controlled osteotomy techniques.

Cortical drills are designed to minimize friction and distribute forces evenly. They effectively reduce stress concentration during bone preparation. This approach allows for precise, gradual, and less traumatic placement.

### Limitations

One of the main limitations of the present study lies in the lack of a precise definition for the dynamic mechanical properties of the mandibular bone, which is critical to enhance the fidelity of dynamic finite element simulations [102]. Currently, most analyses rely on material properties derived from static studies, such as Young’s modulus and Poisson’s ratio, while the dynamic characteristics of a bone during implant insertion are not yet fully understood or accurately modeled. It should be noted that this study made simplified assumptions about the biomechanical behavior of bone tissue. These theories neglected potentially relevant biological and microstructural phenomena that could influence the response to implant insertion. Additionally, the study did not consider different implant microgeometries in relation to transmitted stress [103].

Therefore, further experimental studies and more refined dynamic modeling approaches are essential to improving our understanding of bone behavior during implant insertion and optimizing surgical procedures.

## 5. Conclusions

This study highlights the critical impact of insertion torque on stress distribution within the peri-implant bone surrounding ultra-short implants. Higher insertion torques (75 N·cm) substantially increase peak stresses in both cortical and trabecular bone tissues. In low-density (D4) cortical bone, stress reaches approximately 142 MPa, exceeding the bone’s ultimate strength and raising the risk of microdamage and necrosis. Conversely, moderate insertion torques (around 35 N·cm) facilitate a more favorable stress distribution. This maintains peri-implant stress within physiologically safe limits, thereby reducing the likelihood of microfractures and bone trauma. These results underscore the importance of precise torque control, particularly in low-density bone, to optimize primary stability while minimizing the risk of long-term failure due to mechanical overloading. Proper management of insertion torque is, thus, essential for favorable osseointegration and the long-term success of ultra-short dental implants.

## Figures and Tables

**Table 1:** Mechanical properties of bone block.

Mechanical Properties	Cortical	D2	D4
Density *ρ* (kg/cm^3^)	1.6	0.64	0.32
Young’s modulus, E (GPa)	16	5.5	0.690
Poisson’s ratio, *v*	0.3	0.3	0.3

**Table 2:** Mechanical characteristics of the implant used.

	Young’s Modulus (GPa)	Poisson’s Ratio	Tensile Strength (MPa)
Ti6Al4V	114	0.34	900

## Data Availability

The original contributions presented in the study are included in the article, further inquiries can be directed to the corresponding author.
